# A novel and sensitive method for determining vitamin B3 and B7 by pre-column derivatization and high-performance liquid chromatography method with fluorescence detection

**DOI:** 10.1371/journal.pone.0198102

**Published:** 2018-06-06

**Authors:** Baolei Fan, Jinmao You, Yourui Suo, Chunqi Qian

**Affiliations:** 1 Hubei University of Science and Technology, Xianning, PR China; 2 Northwest Institute of Plateau Biology, Chinese Academy of Sciences, Xining, PR China; 3 Michigan State University, East Lansing, MI, United States of America; Indian Institute of Chemical Technology, INDIA

## Abstract

A new labeling reagent for vitamin analysis, 2-amino-10-ethyl acridine ketone (AEAO), has been synthesized and successfully applied to the analysis of vitamin B3 and vitamin B7 in different tea samples. The reaction of AEAO with vitamins could proceed easily and quickly in the presence of 1-ethyl-3-(3-dimethylaminopropyl)-carbodiimide hydrochloride (EDC) as condensing reagent within 45 min. The derivatives exhibited excellent fluorescence property with excitation and emission wavelengths of 290 nm and 430 nm, respectively. Response surface methodology (RSM) was applied to the optimization of pre-column derivatization. Solid phase extraction with HLB cartridges was used for the extraction and purification of water-soluble vitamins in tea samples. The LODs for vitamin B3 and vitamin B7 were 2.56 and 2.22 ng mL^-1^, respectively. The proposed method was successfully applied to the analysis of vitamin B3 and vitamin B7 in different tea samples. The study provided a highly sensitive method for accurate analysis of trace vitamins from natural products.

## Introduction

**Fig 1:**
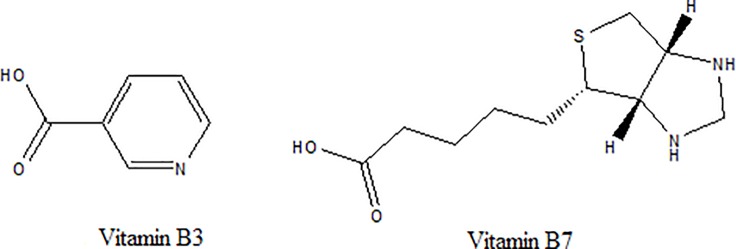
Chemical structure of VB3 and VB7.

Vitamins are vital substances for many organisms [1,2]. Vitamin B3 (nicotinamide and nicotinic acid) is essential to all living cells [3]. It can be used to lower the levels of serum lipids and cholesterol [4]. VB7 (vitamin H and biotin) is a growth promoter at the cellular level. It also affects gene expression through a diverse array of cell signaling pathways [5,6]. Vitamins B3 and B7 (Fig 1.) belong to the B complex water-vitamin families and play specific and vital functions in metabolism. Their deficiency may cause seborrhea, dermatitis, loss of appetite and lassitude [6–8]. As it is well known, vitamins are natural constituents of food, and a well-balanced diet should contain all of the required vitamins. Tea is one of the most popular drinks next to water and used as a popular beverage worldwide since ancient times [9]. Its consumption has been proved to be useful for prevention of many diseases and maintenance of cardiovascular and metabolic health [9–11]. However, there has been limited research on vitamins of tea. That was because the accurate analysis of these compounds was confronted with several challenges: (1) Tea was a complex mixture containing multiple compounds such as phenolics, catechins, polysaccharides and complex thearubigins. (2) Vitamins are the samples in very small quantities, while others substances are at macroscopic levels. (3) Due to their special structure and property, most vitamins are affected by a large number of factors including temperature, moisture, oxygen, light, pro-oxidants, reducing agents and pH [12]. Therefore, it is challenging to develop a sensitive and reliable method suitable for simultaneous determination of different vitamins in a single run.

A number of analytical methods have been developed for vitamins determination, such as volumetric assays [13,14], microbiological assays [15], spectrometric assays [16,17], capillary electrophoresis (CE) [18], gas chromatography (GC) [19], high-performance liquid chromatography (HPLC) with different detection like Ultraviolet–visible spectroscopy (UV-vis) with variable wavelength or diode array detection (DAD) [7,20–25], fluorescence detection (FLD) [26], Electrochemical detection (ED) [27,28], and HPLC/mass spectrometry (LC/MS) [16,29]. Among the above analytical techniques, HPLC methods attract increased attention due to its simplicity, high sensitivity and wide dynamic range. There has been a large number of HPLC-UV studies on the determination of vitamins in foods, beverages, drugs, multivitamin tablets and so on [30–32]. It is not difficult to analyze vitamins in these samples due to their high levels of vitamins. While for plant samples, the analysis of vitamins is difficult, and the extraction procedure is complicated. Thus, there has been limited research on the analysis vitamins in plant samples. In addition, HPLC-UV characterized by lower detection limit cannot satisfy the need of trace constituent determination. Since VB3 and VB7 as water-vitamin with carboxyl group can be easily co-eluted, derivatizing has been widely adopted, which can overcome previous limitations, such as tailing peaks and low detector sensitivity. This method has been recommended for the determination of fatty acids, triterpenic acids etc. in animal and plant samples [33–35], but seldom for vitamins.

In the present study, a new fluorescence reagent 2-amino-10-ethyl-10H- acridin-9-one (AEAO) has been synthesized for the sensitive analysis of vitamins. This compound has shown excellent fluorescence property and it has been used to analyze VB3 and VB7 in tea samples. Response surface methodology (RSM), which is more efficient, requires fewer data and reagent depletion. It provides interfering effects on the response besides factor effects [34]. Therefore, it was applied to optimize pre-column derivatization efficiency. The preparation of crude extracts from plants is also a key step in the qualitative and quantitative analysis of chemical constituents present in the plants. So solid phase extraction (SPE) was applied to remove interfering components. This investigation is the first attempt to apply HPLC-FLD technique in combination with SPE and pre-column derivatization for the sensitive and accurate analysis of vitamin B in tea samples.

## Materials and methods

### 2.1. Instrumentation

The qualitative and quantitative analyses were performed on an Agilent HP 1100 Series high-performance liquid chromatography composed of a vacuum degasser (model G1322A), a quaternary pump (model G1311A), an auto sampler (model G1329A), a column compartment with thermostate (model G1316A), and a DAD detector (G1315B). A fluorescence detector (model G1321A, Agilent, USA) was adjusted at wavelengths of 290 and 430 nm for excitation and emission. Chromatographic separation was achieved on a Hypersil BDS C8 column (200mm×4.6 mm, 5μm i.d., Dalian Elite Analytical Instruments Co., Ltd., China). Solvent A was 15% acetonitrile in water (including 30 mM HCOONH_4_ buffer solution, pH = 3.74), and B was 95% acetonitrile. The gradient condition of mobile phase was as follows: 30%~75% B from 0 to 15min; 75%~100% B from 15 to 20 min. The column was equilibrated with the initial mobile phase for 5 min before the next injection. The flow rate was constant at 1.0 mL min^–1^ and the column temperature was kept at 30 ^o^C. The injection volume was 10 μL. Ultrasound-assisted extractions of vitamins from tea samples were carried out by an ultrasonic cleaner (SB-5200DTD, 40 kHz, Xinzhi Biotech Co., Ningbo, China).

### 2.2. Chemicals

**Fig 2:**
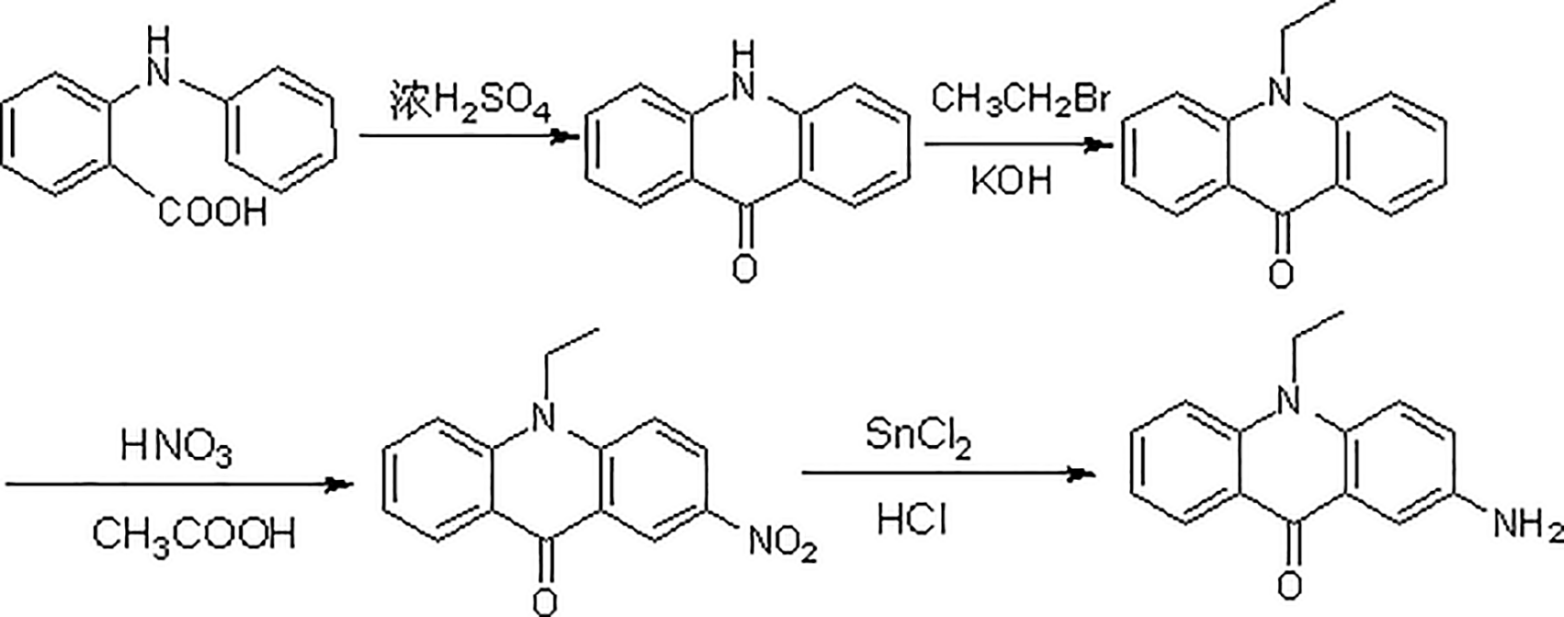
The synthesis procedure of AEAO.

Standards of VB3 and VB7 were of chromatographic grade and purchased from the National Institute for Control of Pharmaceutical and Biological Products (Beijing, China). N-ethyl-N'-[(3-dimethylamino) propyl]carbondiiminehydrochloride (EDC), Pyridine and chloroform were of analytical grade and purchased from Shanghai Chemical Reagent Co. (Shanghai, China). HPLC-grade acetonitrile (ACN) was obtained from Yuwang Company, China. Water was purified on a Milli-Q system (Millipore, Bedford, MA, USA). All other reagents used were also of analytical grade unless otherwise stated. AEAO was synthesized in authors’ laboratory as described in **section 2.3** and Fig 2. Processed tea samples were bought from Hangzhou West Lake Longjing Industry Co.(June, 2011), Ltd. and Guizhou Fanjin Industry Co., Ltd.(July, 2011).

### 2.3. Synthesis of 2-amino-10-ethyl-10H-acridin-9-one (AEAO)

#### 2.3.1. Synthesis of 10-ethyl acridone

Ten grams of acridine ketone was added into a 250-mL three-necked flask containing 100 mL of DMF, and stirred for 20 min. 50 g of KOH was added to dissolved acridine ketone, and heated slowly to 80°C. Twenty-six milliliters of bromoethane with appropriate amount of DMF was titrated drop by drop within one hour. After reacting for one hour at 50°C, the reactant was cooled to room temperature and then poured into 600 mL of distilled water. The precipitated solid was recovered by filtration, washed with water and then dried at 50°C. Yellow transparent flaky crystals can be obtained by recrystallization for three times from ethanol (200 mL×3), with a yield of 85.0%.

#### 2.3.2. Preparation of 2-nitro-10-ethyl acridine ketone

To obtain a solution containing 10 g of 10-ethyl-quinacridone and 200 mL of acetic anhydride, 15 mL mixed solvents of concentrated nitric acid (65%) and glacial acetic acid (1:1.2, v/v) was added dropwise to the solution under mechanical stirring at room temperature. Two hours later, the mixture solution was transferred into 1 L of water. The precipitated solid was recovered by filtration. The pale yellow crystals (yield 85%) was obtained by recrystallization for three times from ethanol (200 mL×3). m.p.:136.2–136.8°C; found (%), C 67.22,H 4.46,N 10.43; calculated (%), C 67.16, H 4.48, N 10.45. IR(KBr): 3459.59 (C-N), 1638.72 (C = O), 1530.49 (NO_2_), 1478.96, 1450.63, 1311.09. APCI, [M+H] ^+^: *m /z* 269.5.

#### 2.3.3. Preparation of 2-amino-10-ethyl acridine ketone (AEAO)

Eight grams of 2-nitro-10-ethyl acridine ketone, 35 g of stannous chloride, 150 mL of concentrated hydrochloric acid and 50 mL of ethanol were mixed in a 250-mL single-necked flask by vigorous stirring. After heating under reflux for two hours, the filtrate was collected by filtration under vacuum, adjusted to pH 8.0, filtrated and dried. Then the crude product was recrystallized two times from ethanol (200 mL×2) to afford orange-red crystals, 80% yield. m.p.: 136.2–136.8°C; found(%), C 75.52,H 5.60,N 11.79;calculated (%),C 75.63, H 5.58, N 11.79. IR (KBr): 3458.71 (C-N), 3326.29 (NH_2_), 1632.38 (C = O), 3401.79, 1501.36, 1295.59, 1188.29. APCI, [M+H] ^+^: *m/z* 239.7.

### 2.4. Preparation of standard solutions

The preparation of standard solutions used in our study was based on a combination of vitamin hydrolysis according to the literatures[24,32]. VB3 and VB7 at a concentration of 1.5×10^−4^ mol mL^-1^ were prepared in water containing 0.1% (v/v) HCl. The working standard solutions for calibration curves were obtained by diluting the mixed stock solution with acetonitrile at different concentrations. EDC solution (0.2 mol L^–1^) and AEAO solution (1.0×10^−2^ mol L^–1^) were prepared in acetonitrile. The corresponding low concentration solution was obtained by diluting the stock solution with acetonitrile. When not in use, all solutions were stored at 4 ^o^C in a refrigerator until HPLC analysis.

### 2.5. Sample preparation

#### 2.5.1. Ultrasonic-assisted extraction

The prepared sample (50 mg) was weighed in a 5-mL glass centrifuge tube and then mixed with 3 mL of 0.01 mol L^–1^ HCl. Extraction was performed with ultrasonication at 90 ^o^C for 30 min. Sample was centrifuged at 4000 r/min for 10 min. Then the supernatant was collected, and 2 mL of 0.01 mol L^–1^ HCl was added into the residue for further extraction. The secondary supernatants were collected and used for further analysis.

#### 2.5.2. Solid phase extraction (SPE)

The extracts of tea samples consist of many components which may cause chromatographic interferences with vitamins. We used the Waters Oasis HLB (200 mg, 6 mL) cartridges to remove most of the interfering components. First, we flushed the stationary phase with 5 mL of methanol and 5 mL of water (0.1%, HCl) to activate the stationary phase, respectively, then 2 mL of tea extract was added. The sample was eluted with 10 mL of mixed solvent containing water (0.1%, HCl)/methanol (9:1, v/v). The eluents were collected in a bottle and evaporated to dryness. The residue was dissolved in acidified water (0.1%, HCl) solution.

**Fig 3:**
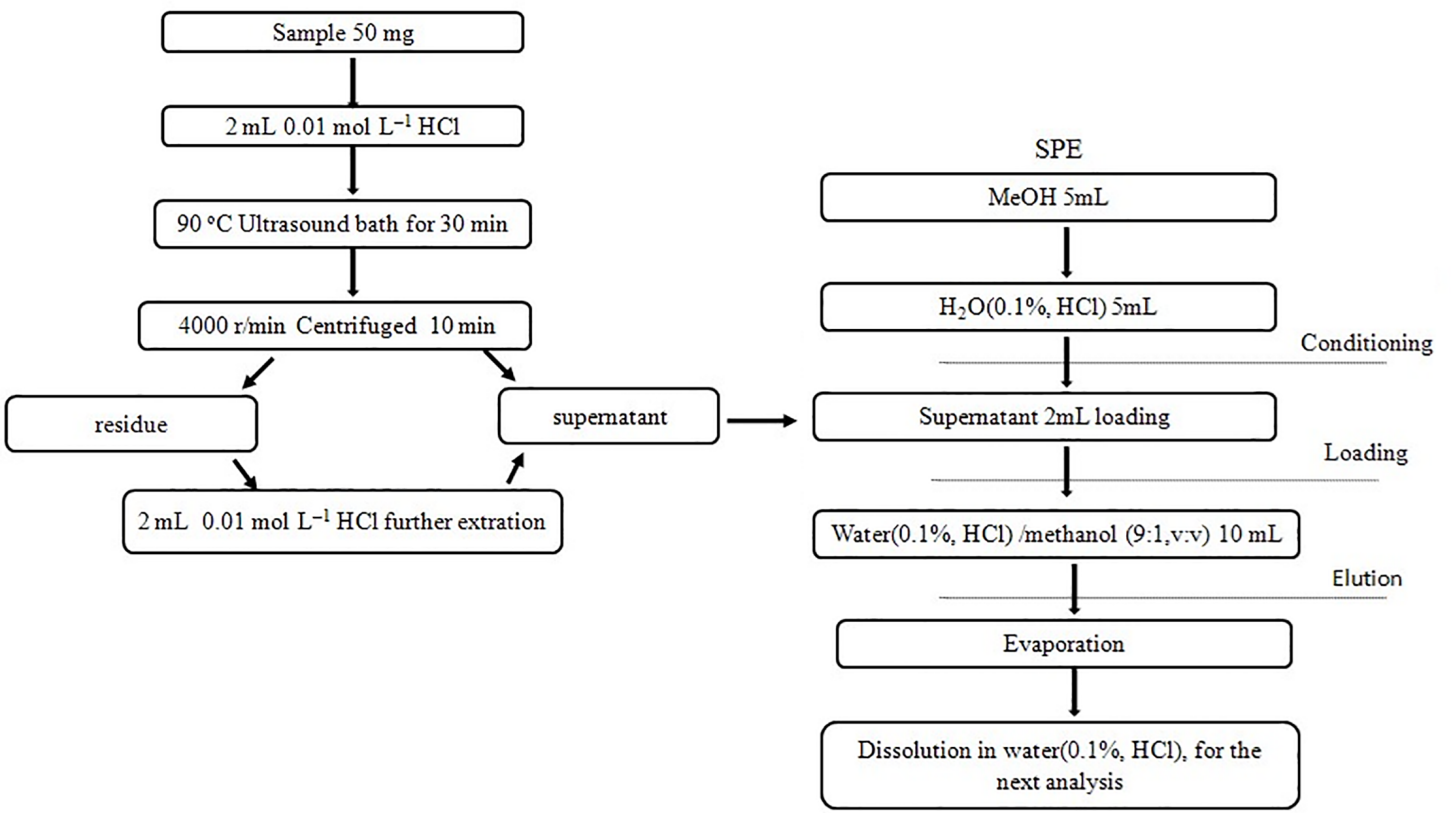
The extraction process of tea samples.

The whole sample preparation procedures were carried out in darkness. The extraction process is depicted in Fig 3.

### 2.6. Box-Behnken design for pre-column derivatization optimization

The software Design Expert (Trial Version 7.0.3, Stat-Ease Inc., Minneapolis, MN, USA) was employed for experimental design, data analysis and model building. Box-Behnken design with three variables was used to determine the response pattern and then to establish a model. Three variables with three levels of each variable used for derivatization optimization were derivatization temperature (^o^C, X_1_), dosage of EDC (μL, X_2_) and time (min, X_3_), and the peak area (VB3 and VB7) was taken as the response, Y, which reflects the detector response. The symbols and levels are presented in Table 1.

**Table 1:** Box-Behnken design and observed response.

Run	Independent variable			Response (Peak area)
	X_1_ (Temperature, °C)	X_2_(EDC, μL)	X_3_ (Time, min)	
1	80.00(0)	160.00(+1)	30.00(-1)	456
2	60.00(-1)	60.00(-1)	75.00(0)	398
3	80.00(0)	110.00(0)	75.00(0)	586
4	100.00(+1)	110.00(0)	120.00(0)	405
5	100.00(+1)	110.00(0)	30.00(-1)	428
6	80.00(0)	60.00(-1)	120.00(+1)	250
7	60.00(-1)	110.00(0)	120.00(+1)	456
8	80.00(0)	110.00(0)	75.00(0)	582
9	100.00(+1)	60.00(-1)	75.00(0)	525
10	80.00(0)	110.00(0)	75.00(0)	567
11	80.00(-1)	110.00(0)	75.00(0)	589
12	100.00(+1)	160.00(+1)	75.00(0)	452
13	60.00(-1)	110.00(0)	30.00(-1)	386
14	80.00(0)	60.00(-1)	30.00(-1)	521
15	80.00(0)	110.00(0)	75.00(0)	549
16	60.00(-1)	160.00(+1)	120.00(+1)	421
17	80.00(0)	160.00(+1)	30(+1)	271

Based on the experiment data, regression analysis was performed and fitted into the empirical second order polynomial model, based on the following equation:

$$Y=\beta_{0}+\sum_{i=1}^{3}\beta_{i}X_{i}+\sum_{i=1}^{3}\beta_{ii}X_{ii}+\sum_{i=1}^{2}\sum_{j=i+1}^{3}\beta_{ij}X_{jj}$$

where Y represents the response variable, β_0_ is a constant, β_i_, β_ii_, and β_ij_ are the linear, quadratic and interactive coefficients, respectively.

### 2.7. Derivatization procedure

**Fig 4:**
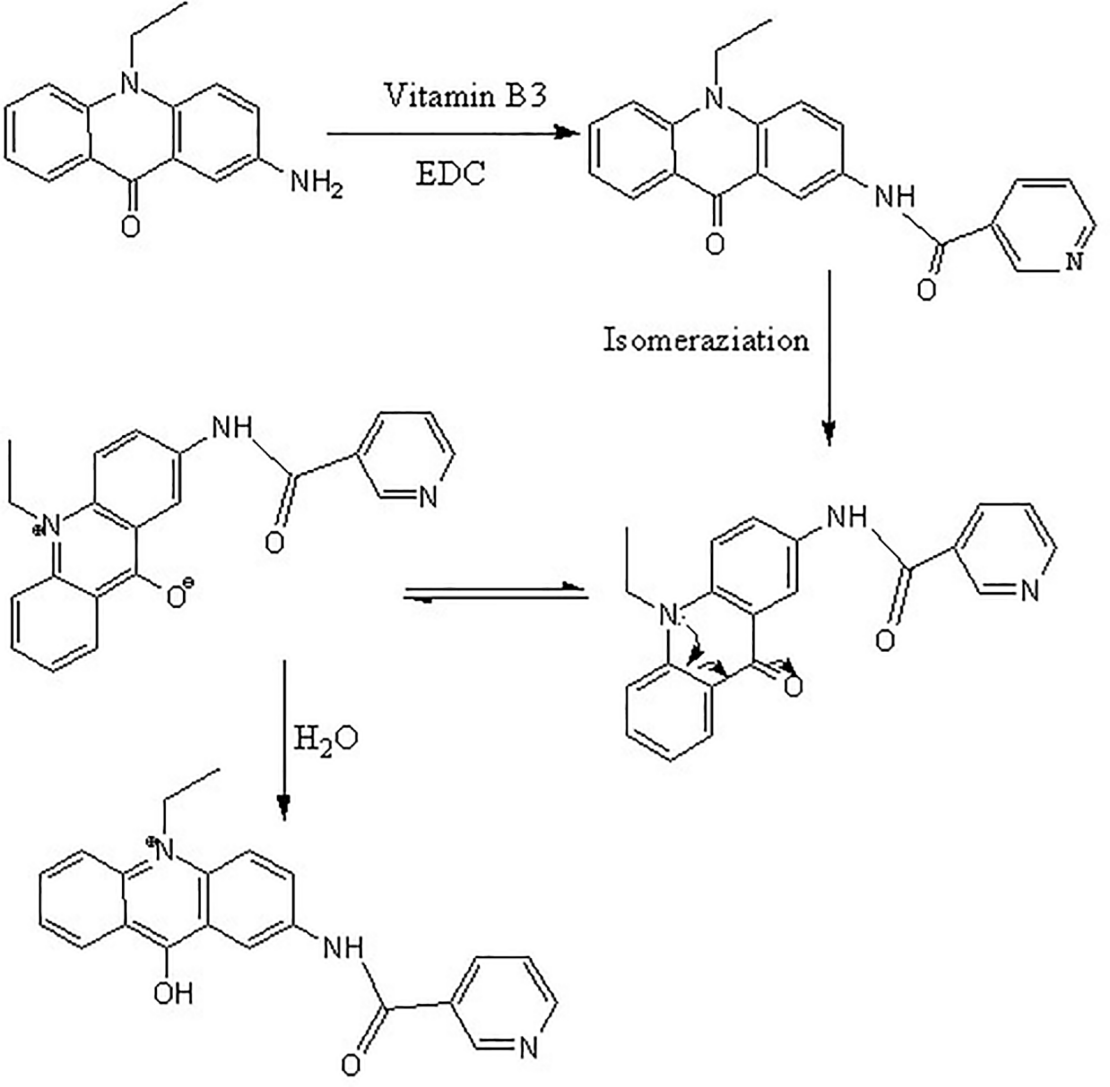
Scheme of derivation reaction of AEAO with VitaminB7.

The derivatization procedure is shown in Fig 4. According to the optimal derivatization conditions obtained from RSM, 10 uL of derivatizing reagent solution and ECD were added to a solution containing a standard vitamin mixture or sample in a 2-mL vial. The vial was sealed and allowed to react in a water bath. After the reaction was completed, the mixture was cooled to room temperature, then an appropriate volume of acetonitrile solution was added to dilute the derivatization solution. The diluted solution was syringe filtered using a 0.22-mm nylon filter and injected directly for HPLC analysis (10 μL).

### 2.8. Method validation

The validity of this analytical method was fully evaluated by linearity, recovery, limit of detection (LOD), limit of quantification (LOQ), as well as the intra-day and inter-day variability and precision. (LOQ is the concentration when the signal-to-noise ratio is 10:1 and LOD is the concentration when the signal-to-noise ratio is 3:1.) Retention time reproducibility was evaluated in the plasma. Recovery was calculated as the ratio of the signal area of extracted substance dissolved in matrix spike over the signal area of matrix spike. Precision was determined by the coefficient of variation between the concentrations measured among different repetitions. Method linearity range was validated through the correlation coefficient observed for the calibration curves. Each standard concentration was analyzed 3 times by HPLC and the standard curves of concentrations were plotted against peak heights. Three concentrations of VB3 were prepared at 5.0, 10.0 and 1.5 ng mL^-1^; three concentrations of VB7 were prepared at 4.0, 8.0 and 10.0 ng mL^-1^.

## Results and discussion

### 3.1. Stability and fluorescence spectral properties of AEAO

AEAO is stable in common organic solvents and water. It could also be placed at room temperature for 6 months without any decomposition. Strong basic or acidic conditions should be avoided because AEAO may hydrolyze under such conditions. It is stable during the process of derivatization reaction. The derivatives were placed in the refrigerator at 4 ^o^C for 3 days with the normalized peak areas deviations of <2.6%. The maximum excitation and emission wavelengths for them were 290 and 430 nm, respectively.

### 3.2. Optimization of extraction

Vitamin B is stable in weak acid solution and prone to be oxidized under neutral or alkaline solution. Because sulfuric acid (H_2_SO_4_) and phosphoric acid (H_3_PO_4_) are oxidants, hydrochloric acid (HCl) was chosen for extraction solution. The influence of HCl concentrations on extraction was studied with HCl concentrations ranging from 0.001 to 0.1 mol L^–1^. Results showing the maximum yield of vitamin were obtained when the solvent was 0.01 mol L^–1^ HCl. Ultrasonication extraction was readily applied in our experiment.

Tea samples have complex composition, and vitamins only exist at low level. Some components, such as phenols, amino acids, and saccharides etc. may interfere with the qualitative and quantitative analysis. So solid phase extraction is necessary prior to HPLC to remove interfering components. The recovery was analyzed by an internal standard method. The result indicated HLB cartridge as the solid phase extraction tool was useful for preconcentration and recovery of water soluble vitamins in the tea samples, leading to minimal loss of vitamins.

### 3.3. Optimization of derivatization conditions

Optimization of reaction condition is a key step for pre-column derivatization. The derivatization scheme of AEAO with typical VB7 was shown in Fig 4. In this study, different condensing agents used in the derivatization reaction were evaluated, including 4-dimethylaminopyridine (DMAP), l-hydroxy-benzotriazole (HOBt), K_2_CO_3_ and EDC. The results indicated that EDC was the best basic catalyst and gave the highest detection responses. The ECD dosage, derivatization temperature and time were chosen for further optimization based on its significant effect on derivatization yields. Based on the molar ratio of AEAO to vitamins, when the quantity of AEAO was 10 times the standard, the derivative to yield was highest.

The condition for AEAO derivatization was optimized according to the Box-Behnken design. Based on the preliminary test to optimize the main derivatization conditions, including derivatization temperature, derivatization time and EDC dosage, the coded and uncoded independent variables were used in the RSM design. Their respective levels and experimental results were given in Table 1. For the statistical analysis of experimental results, enter method was used to calculate the estimated coefficients of the polynomial functions of response surfaces. The predicted second-order polynomial model was:

$$Y=57.48+2.04X_{1}-1.36X_{2}-1.35X_{3}-2.05X_{1}X_{2}-2.33X_{1}X_{3}+2.13X_{2}X_{3}-8.02X_{1}^{2}-4.92X_{2}^{2}-7.59X_{3}^{2}$$

The analysis of variance (ANOVA) for the experimental results was listed in Table 2. A “Model F-value” of 30.16 implied that the model was significant, and there was only a 0.01% chance that a “Model F-Value” of this large could occur due to noise. Values of “Prob > F” less than 0.0500 indicated model terms were significant. In this case A, B, AB, A_2_, B_2_, C_2_ were significant model terms. The value of R^2^ (0.9856) indicated that the model adequately represented the real relationship between the parameters chosen. A “Lack of Fit F-value” of 3.00 implied that “the Lack of Fit” was not significant (P>0.05), and that that this model was sufficiently accurate for predicting the relevant responses. Coefficient of variation (C.V. %) less than 3.07% indicated that the model had a better precision and reliability.

**Table 2:** Estimated regression coefficients for the quadratic polynomial model and ANOVA for the experimental results in the optimization of flavonoids extractions.

Source	Sum of Squares	df	Mean of Squares	F Value	P-value (Prob>F)
Model	802.24	9	89.14	6.04	0.0135
A-T	33.21	1	33.21	2.25	0.0171
B-V	14.85	1	14.85	1.01	0.0347
C-t	14.58	1	14.58	0.99	0.0354
AB	16.81	1	4.69	5.95	0.0499
AC	21.62	1	0.33	0.42	0.5379
BC	18.06	1	1.92	2.43	0.1627
A^2^	270.49	1	270.49	18.33	< 0.0001
B^2^	101.71	1	101.71	6.89	0.0034
C^2^	242.56	1	242.56	16.43	0.0048
Residual	5.52	7	14.76		
Lack of Fit	3.82	3	30.80	11.30	0.0201
Pure Error	1.70	4	2.73		
Cor Total	905.55	16	C.V. %		
R-Squared	0.9859		Adeq	6.318	
Adj R-Squared	0.7392		Precision	10.826	
Pred R-Squared	0.6516		C.V.%	3.07	

**Fig 5:**
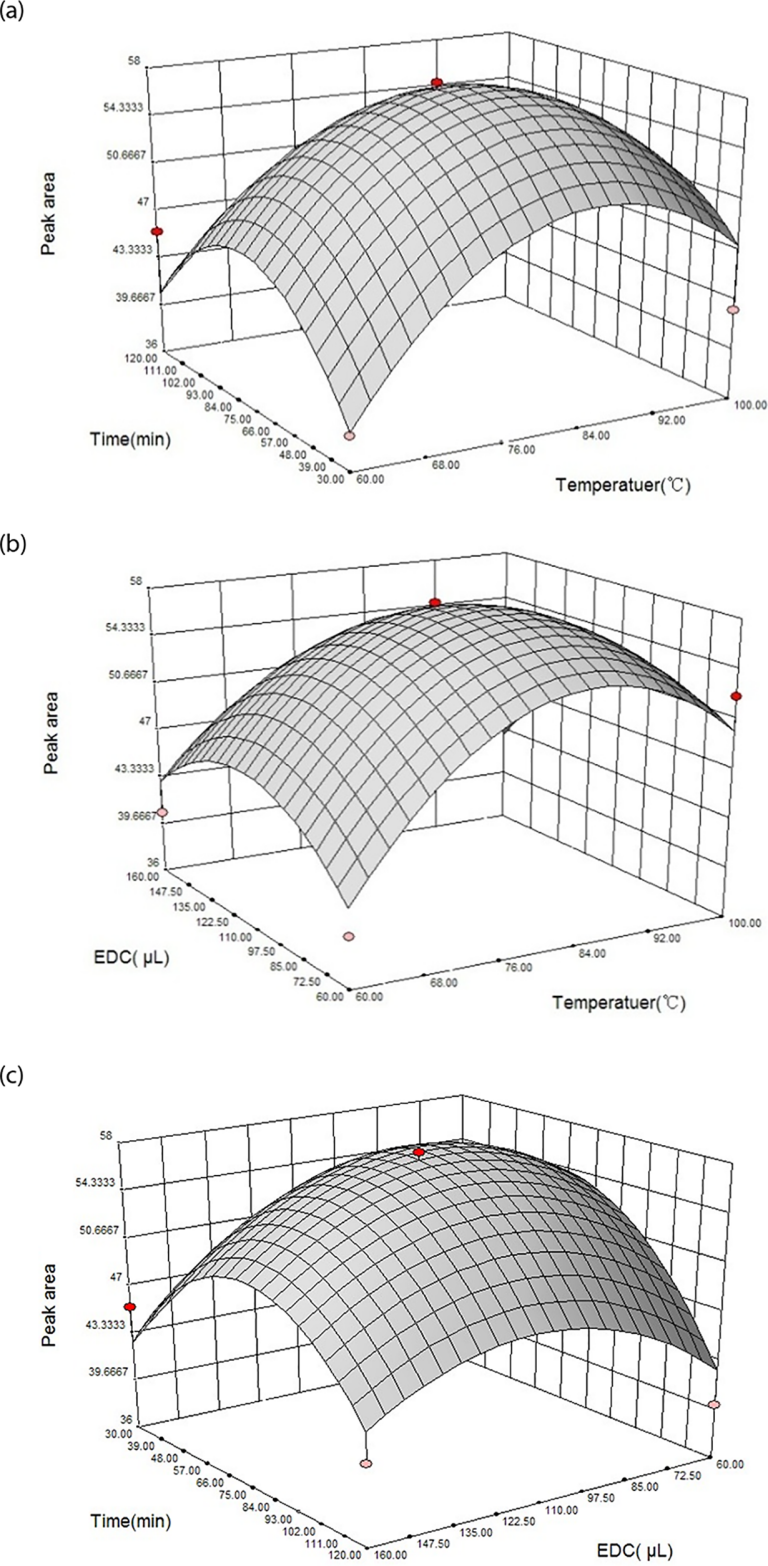
The 3D response surface of peak area affected by varying derivatization temperature and time(a), varying derivatization temperature and EDC dosage (b), derivatization time and EDC dosage (c).

Three-dimensional (3D) response surface provided an effective way to visualize the relationship between responses and variables, the interactions between two test variables, as well as the optimum extraction conditions (see Fig 5). Fig 5A depicted the interactive effect of derivatization time and derivatization temperature on the response (peak area of VB) at the fixed value of EDC dosage. The increase of temperature can significantly enhance the response: the peak area of VB increased rapidly, reached a maximum value, followed by a decline with further increase in temperature. The effect of derivatization temperature and dosage of EDC on the peak area of VB at the fixed value of the derivatization time was shown in Fig 5B. Both EDC dosage and temperature have positive effect on the response. Fig 5C described the interactive effect of EDC dosage and derivatization time on the content of peak area of VB at the fixed value of derivatization temperature.

The optimal conditions obtained from the model were as follows: derivatization time of 44.8 min, derivatization temperature at 89.6°C and 91 μL of EDC. As a compromise, derivatization conditions were defined to be 90μL of EDC, derivatization temperature at 90°C, derivatization time of 45 min. Under optimized conditions, the model predicted the value of derivatization yield (peak area of VB3 and VB7) would be 627. To test validity of response surface analysis method, derivatization was carried out under the same conditions, and the derivatization yield was 625 (*n* = 3). The good correlation (R^2^ = 0.9856) between these results confirmed that the response model was adequate to reflect the expected optimization.

### 3.4. Chromatographic separation

**Fig 6:**
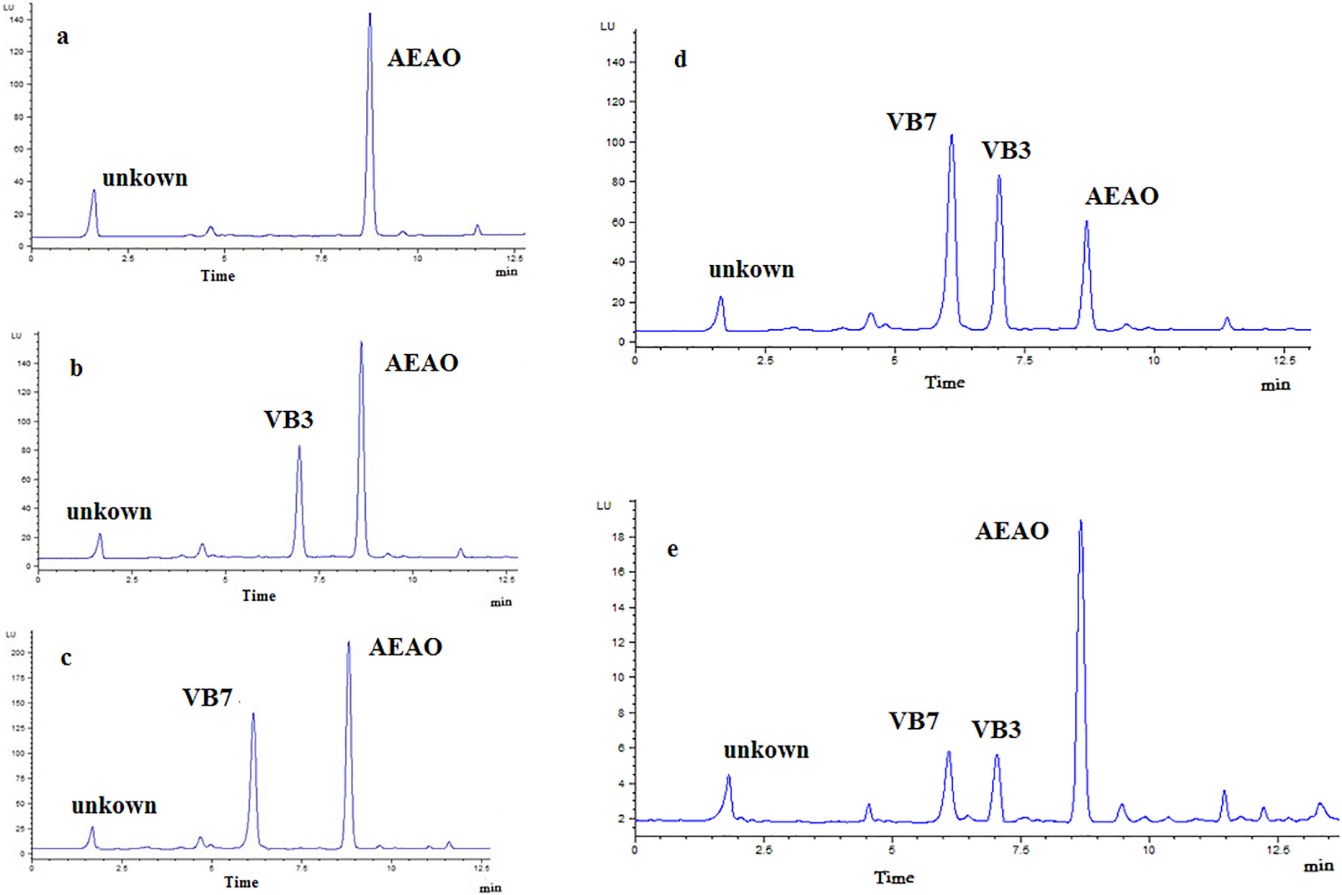
The representative chromatograms for blank (a) spiked blank with standard VB3(b),VB7(c), VB3 and VB7(d), and tea samples (e).

Chromatographic separation is a key step in HPLC analysis. The separation of VB3 and VB7 derivatives was investigated to obtain satisfactory result within the shortest amount of time. The resolutions of these compounds were tested and compared with different reversed-phase conditions using a variety of analytical columns such as Hypersil BDS C8 column (200mm×4.6mm, 5μm), Hypersil C18 (250mm×4.6mm, 5μm), and Eclipse XDB C8 column (150mm×4.6mm, 5μm). Finally, the Hypersil BDS C8 column (200mm×4.6 mm, 5μm) was chosen due to its good separation efficiency. In this experiment, 15% acetonitrile in water containing 30 mM HCOONH_4_ buffer solution (pH = 3.74) was selected as the mobile phase, and HCOONH_4_ buffer solution provided better separation and resolution for target peaks. The study showed the acetonitrile/water/ammonium formate mobile phase resulted in better performances in terms of peak shape for vitamins. Representative chromatograms for the standard and samples analytes are shown in Fig 6A–6E.

### 3.5. Method evaluation

Validation of the analytical method was evaluated by linearity, limit of detection (LOD), limit of quantitation (LOQ), accuracy (recovery studies) and precision (repeatability and intermediate precision) according to United States Food and Drug Administration (FDA) guidelines [36].

Linearity data was generated by plotting the peak areas versus concentrations in the range of 1.5~100 ng mL^−1^and 0.5~80 ng mL^−1^ for standard VB3, and VB7, respectively. The correlation coefficients were found to be 0.9992 and 0.9997, indicating excellent linearity of the analytes. The LOD and LOQ were calculated at the signal-to-noise (S/N) ratio of 3 and 10, respectively, which were presented in Table 3.

**Table 3:** Linear range, correlation coefficient, precision, accuracy, quantitative limits, detection limits and repeatability.

Vita min	RT (min)	Linear range (ng mL^-1^)	Correlation coefficient	Precision (%) n = 3		Accuracy (%) n = 3		LOD (ng mL^-1^)	LOQ (ng mL^-1^)	RSD (%)	
				Intra-day	Inter-day	Intra-day	Inter-day	Method	Method	Retention time	Peak area
VB3	7.01	1.5–100	0.9992	3.48	3.96	92.16	90.23	4.76	14.28	0.02	1.60
VB7	6.24	0.2–80	0.9997	4.75	4.09	104.06	101.12	2.56	7.68	0.015	1.89

The accuracy and precision (intra-day and inter-day) were investigated by spiking VB3 andVB7 of three different concentrations (1.5, 15 and 75 ng mL^−1^) into tea samples. The spiked samples were derivatized according to the procedure described in **Section 2.7** and analyzed by the proposed method. All analyses were carried out in triplicate. Precision (inter- and intra-day) obtained RSDs were ≤4.75%. The intra-day accuracy ranged from 92.16% to 104.06%, while the inter-day accuracy ranged from 90.23% to 101.12%, indicating the little loss during SPE procedure and an excellent accuracy of the proposed method.

### 3.6. Analysis of VB3 and VB7 in the tea

The proposed method was applied to analysis of VB3 and VB7 in different tea samples. Standard solution was injected after every 5 samples to compensate for the possible deviation in retention times. Fig 5 shows a representative chromatogram of VB standard solution and a chromatogram of VB3 and VB7 in the tea. The contents of VB3 and VB7 in different tea species are summarized in Table 4. As the results reported in Table 4, the contents of VB3 were higher than those of VB7 in the tea samples. Rattan tea has the highest content of VB3 and VB7. The VB3 content in green tea was lower than those of red tea and black tea, while VB7 content in green tea was higher than those in red tea and black tea.

**Table 4:** Main content of VB3 and VB7 in the tea.

Tea	species	VB3		VB7	
		Content (μg g^-1^)	RSD (%,n = 5)	Content (μg g^-1^)	RSD (%,n = 5)
Green Tea	Longjing	110.51	1.0	20.01	0.8
Green Tea	Cuifeng1	137.65	1.4	25.01	1.9
Green Tea	Cuifeng2	129.07	0.9	18.97	1.1
Green Tea	Maofeng	90.11	1.5	11.55	1.2
Red Tea	Gongfu	204.39	1.3	2.16	2.5
Black Tea	Tianjian	180.32	0.8	2.77	2.3
Black Tea	Fuzhuan1	270.96	1.6	4.75	2.0
Black Tea	Fuzhuan2	248.07	0.8	6.09	1.6
Black Tea	Qianliang	289.35	0.7	7.92	0.9
Ratten Tea	Guizhou	382.61	1.1	30.98	0.7

### 3.7. Comparisons of the proposed method with previously reported methods

To demonstrate the merit of the present method, this study was compared with some previous analytical methods for vitamins. The results were listed in Table 5. As shown in Table 5, the sensitivity of this method was much higher than those of CE, HPLC-UV, HPLC-DAD and LC–MS/MS methods, almost the same as the HPLC-ED technique, in which the analyte must have the property of oxidation-reduction. The HPLC sensitivity when using AEAO as derivatizing reagent was higher than those applying traditional technique. In addition, the proposed method also shows the merit of less sample consumption. The sample amount used in this method is only 50 mg, therefore, it can be well applied in the analysis of vitamins in precious samples.

**Table 5:** Comparisons of the proposed method with previously reported methods.

Analytes	Vitamin	Extraction	Method	LOD	LOQ	Refences
Drink	water-soluble	in-capillary enzyme reaction	CE	0.1–1.5 μg mL^-1^	—	[31]
Human rine	water-soluble	SPE (Sep-Pak C18)	HPLC-UV	0.33–5.4μg mL^-1^	—	[32]
Italian pasta	water-soluble	Hydrolysis-extraction	LC-MS/MS	0.5–5μg mL^-1^	2–15μgmL^-1^	[29]
Milk formula	fat -soluble	Semi-micro liquid-liquid extraction	HPLC-UV	0.003μg mL^-1^VA 0.05μg mL^-1^ VE	— —	[21]
Multivitamin syrup	water-soluble	Acid digestion (*o*-phosphoric acid)	HPLC-DAD	0.1–0.7μg mL^-1^	0.3–2.3μg mL^-1^	[21]
pharmaceutical preparation	water-soluble	Immobilized enzyme	IMER-HPLC	0.05μg mL^-1^	—	[28]
Apple juices	water-soluble	Acid digestion and enzymatic hydrolysis	HPLC-ED-UV	—	0.11-62.ng mL^-1^	[14]
Tea	water-soluble	Acid digestion and SPE	HPLC-FLD	2.56,4.76 ng mL^-1^	12.22,7.15ng mL^-1^	*

* The present study.

## Conclusions

In the present study, a novel fluorescent labeling reagent (AEAO) has been well synthesized and successfully applied to determine the amount of VB3 and VB7 in tea samples. The synthesis procedure is simple and the synthesized AEAO is stable. The SPE extraction was useful for preconcentration and recovery of vitamins with minimum loss of vitamins. The derivatization conditions were optimized by RSM. The results showed that the precision (inter- and intra-day) of the method (RSDs) were ≤4.75%. The intra-day accuracy ranged from 92.16% to 104.06%, while the inter-day accuracy ranged from 90.23% to 101.12%, indicating an excellent accuracy of the proposed method. The proposed method is sensitive and selective enough to be applied to the determination of vitamins in other samples, including food, vegeterbles as well as furits. The method provided a sensitive, efficient and convenient alternative for simultaneous analysis of the biological samples with trace content of bioactive components.
